# *catena*-Poly[[zinc-bis­(μ-2-sulfido-1*H*-benzimidazol-3-ium-5-carboxyl­ato)-κ^2^ *O*:*S*;κ^2^ *S*:*O*] trihydrate]

**DOI:** 10.1107/S1600536811006532

**Published:** 2011-03-05

**Authors:** Ya-Ping Li, Da-Jun Sun, Hu Zang, Li-Ying Han, Guan-Fang Su

**Affiliations:** a Department of Ophthalmology, the Second Hospital of Jilin University, Changchun 130041, People’s Republic of China; b Department of Vascular Surgery, the China–Japan Union Hospital of Jilin University, Changchun 130033, People’s Republic of China; c Department of Orthopedics, the China–Japan Union Hospital of Jilin University, Changchun 130033, People’s Republic of China; d Department of Gynecology, The Second Hospital of Jilin University, Changchun 130041, People’s Republic of China

## Abstract

In the title compound, {[Zn(C_8_H_5_N_2_O_2_S)_2_]·3H_2_O}_*n*_, the Zn^II^ atom, lying on a twofold rotation axis, is four-coordinated by two S atoms and two O atoms from four 2-sulfido-1*H*-benzimidazol-3-ium-5-carboxyl­ate (H_2_mbidc) ligands in a distorted tetra­hedral geometry. Two H_2_mbidc ligands bridge two Zn^II^ atoms, generating a double-chain along [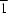
01]. Adjacent chains are linked by N—H⋯O and O—H⋯O hydrogen bonds, forming a three-dimensional supra­molecular network. One of the two water molecules also lies  on a twofold rotation axis.

## Related literature

For coordination polymers with helical chain structures, see: Chen & Liu (2002 ▶); Cui *et al.* (2003 ▶); Hu *et al.* (2008 ▶); Ngo & Lin (2002 ▶); Xiao *et al.* (2007 ▶); Yan *et al.* (2005 ▶).
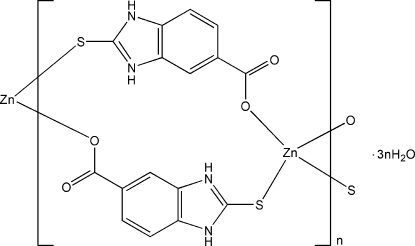


## Experimental

### 

#### Crystal data

[Zn(C_8_H_5_N_2_O_2_S)_2_]·3H_2_O
*M* *_r_* = 505.86
Monoclinic, 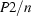

*a* = 8.031 (1) Å
*b* = 9.732 (3) Å
*c* = 12.436 (7) Å
β = 96.584 (9)°
*V* = 965.6 (6) Å^3^
*Z* = 2
Mo *K*α radiation
μ = 1.54 mm^−1^
*T* = 293 K
0.20 × 0.18 × 0.15 mm

#### Data collection

Bruker APEXII CCD diffractometer
Absorption correction: multi-scan (*SADABS*; Sheldrick, 1996) ▶ *T*_min_ = 0.749, *T*_max_ = 0.802
4749 measured reflections
1710 independent reflections
1267 reflections with *I* > 2σ(*I*)
*R*_int_ = 0.049

#### Refinement

*R*[*F* ^2^ > 2σ(*F* ^2^)] = 0.042
*wR*(*F* ^2^) = 0.099
*S* = 0.98
1710 reflections
137 parameters
H-atom parameters constrained
Δρ_max_ = 0.42 e Å^−3^
Δρ_min_ = −0.28 e Å^−3^

### 

Data collection: *APEX2* (Bruker, 2007 ▶); cell refinement: *SAINT* (Bruker, 2007 ▶); data reduction: *SAINT*; program(s) used to solve structure: *SHELXS97* (Sheldrick, 2008 ▶); program(s) used to refine structure: *SHELXL97* (Sheldrick, 2008 ▶); molecular graphics: *SHELXTL* (Sheldrick, 2008 ▶) and *Mercury* (Macrae *et al.*, 2006 ▶); software used to prepare material for publication: *SHELXTL*.

## Supplementary Material

Crystal structure: contains datablocks global, I. DOI: 10.1107/S1600536811006532/hy2396sup1.cif

Structure factors: contains datablocks I. DOI: 10.1107/S1600536811006532/hy2396Isup2.hkl

Additional supplementary materials:  crystallographic information; 3D view; checkCIF report

## Figures and Tables

**Table 1:** Hydrogen-bond geometry (Å, °)

*D*—H⋯*A*	*D*—H	H⋯*A*	*D*⋯*A*	*D*—H⋯*A*
N1—H1⋯O1*W*	0.86	1.88	2.738 (5)	174
N2—H2⋯O1^i^	0.86	1.98	2.812 (4)	163
O1*W*—H1*A*⋯O2^ii^	0.84	2.21	2.907 (4)	140
O2*W*—H2*A*⋯O1	0.82	2.02	2.837 (4)	177

Symmetry codes: (i) 
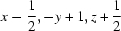
; (ii) 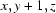.

**Fig. 1.**
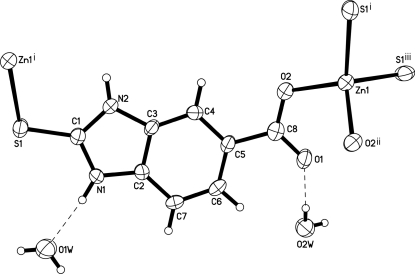
The asymmetric unit of the title compound. Displacement ellipsoids are drawn at the 50% probability level. H atoms have been omitted for clarity. Dashed lines denote hydrogen bonds. [Symmetry codes: (i) 1 - x, 1 - y, 2 - z; (ii) 3/2 - x, y, 3/2 - z; (iii) 1/2 + x, 1 - y, -1/2 + z.]

**Fig. 2.**
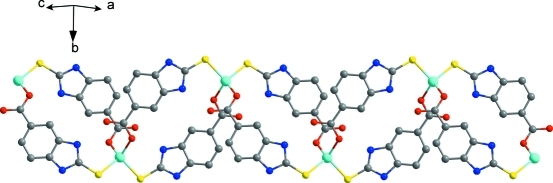
A view of the double-chain structure in the title compound.

## supplementary crystallographic information

### Comment

In recent years, the synthesis of novel coordination polymers with helical structures has attracted much attention owing to the fundamental role of helicity in biology and their potential utilization in advanced materials (Cui *et al.*, 2003; Ngo & Lin, 2002; Yan *et al.*, 2005). In general, the V-shaped organic ligands have already been proven to be efficient for the generation of helical complexes (Chen & Liu, 2002; Hu *et al.*, 2008; Xiao *et al.*, 2007). 2-Mercapto-1*H*-benzo[*d*]imidazole-5-carboxylic acid (H_3_mbidc) is a rigid V-shaped ligand, in which the S atom can coordinate to a variety of metal ions and the carboxylate group can adopt rich coordination modes, meeting the requirements of the coordination geometries of metal ions in assembly process. We selected H_3_mbidc as a bridging ligand and Zn^II^ ion as a metal center, generating a new double-chain coordination polymer, whose structure is reported here.

In the title compound (Fig. 1), the Zn^II^ atom is four-coordinated by two S atoms and two carboxylate O atoms from four individual H_2_mbidc ligands in a distorted tetrahedral coordination geometry. The Cd—O and Cd—S bond lengths are 1.987 (3) and 2.3159 (12) Å. Each H_2_mbidc ligand bridges two neighboring Zn^II^ atoms, generating a double-chain (Fig. 2). Furthermore, N—H···O and O—H···O hydrogen bonds (Table 1) link the chains together, resulting in a supramolecular structure.

### Experimental

A mixture of H_3_mbidc (0.971 g, 5 mmol), NaOH (0.4 g, 10 mmol) and ZnCl_2_ (1.36 g, 10 mmol) in water (50 ml) was boiled for 20 min with stirring. Then the mixture was cooled to room temperature. The resulting solution was filtered and allowed to stand. After a week, colorless crystals of the title compound were obtained.

### Refinement

H atoms on C and N were positioned geometrically and refined as riding atoms, with C—H = 0.93 and N—H = 0.86 Å and *U*_iso_(H) = 1.2*U*_eq_(C,*N*). H atoms of water molecules were located in a difference Fourier map and refined as riding atoms, with *U*_iso_(H) = 1.5*U*_eq_(O).

### Crystal data

**Table:**

[Zn(C_8_H_5_N_2_O_2_S)_2_]·3H_2_O	*F*(000) = 516
*M**_r_* = 505.86	*D*_x_ = 1.740 Mg m^−^^3^
Monoclinic, *P*2/*n*	Mo *K*α radiation, λ = 0.71073 Å
Hall symbol: -P 2yac	Cell parameters from 4749 reflections
*a* = 8.031 (1) Å	θ = 1.3–26.0°
*b* = 9.732 (3) Å	µ = 1.54 mm^−^^1^
*c* = 12.436 (7) Å	*T* = 293 K
β = 96.584 (9)°	Block, colorless
*V* = 965.6 (6) Å^3^	0.20 × 0.18 × 0.15 mm
*Z* = 2	

### Data collection

**Table:**

Bruker APEXII CCD diffractometer	1710 independent reflections
Radiation source: fine-focus sealed tube	1267 reflections with *I* > 2σ(*I*)
graphite	*R*_int_ = 0.049
φ and ω scans	θ_max_ = 25.0°, θ_min_ = 2.1°
Absorption correction: multi-scan (*SADABS*; Sheldrick, 1996)	*h* = −9→8
*T*_min_ = 0.749, *T*_max_ = 0.802	*k* = −9→11
4749 measured reflections	*l* = −14→14

### Refinement

**Table:**

Refinement on *F*^2^	Primary atom site location: structure-invariant direct methods
Least-squares matrix: full	Secondary atom site location: difference Fourier map
*R*[*F*^2^ > 2σ(*F*^2^)] = 0.042	Hydrogen site location: inferred from neighbouring sites
*wR*(*F*^2^) = 0.099	H-atom parameters constrained
*S* = 0.98	*w* = 1/[σ^2^(*F*_o_^2^) + (0.0471*P*)^2^ + 0.5228*P*] where *P* = (*F*_o_^2^ + 2*F*_c_^2^)/3
1710 reflections	(Δ/σ)_max_ < 0.001
137 parameters	Δρ_max_ = 0.42 e Å^−^^3^
0 restraints	Δρ_min_ = −0.28 e Å^−^^3^

### Fractional atomic coordinates and isotropic or equivalent isotropic displacement parameters (Å^2^)

**Table:**

	*x*	*y*	*z*	*U*_iso_*/*U*_eq_	
Zn1	0.7500	0.20862 (7)	0.7500	0.0295 (2)	
S1	0.46579 (13)	0.94066 (11)	1.22275 (9)	0.0366 (3)	
O1	0.9750 (3)	0.4579 (3)	0.7829 (2)	0.0360 (7)	
O2	0.8006 (3)	0.3368 (3)	0.8738 (2)	0.0323 (7)	
N1	0.6753 (4)	0.9126 (3)	1.0706 (2)	0.0283 (8)	
H1	0.6905	0.9997	1.0655	0.034*	
N2	0.5899 (4)	0.7152 (3)	1.1261 (2)	0.0298 (8)	
H2	0.5423	0.6551	1.1630	0.036*	
C1	0.5795 (4)	0.8514 (4)	1.1382 (3)	0.0266 (9)	
C2	0.7452 (4)	0.8136 (4)	1.0112 (3)	0.0258 (9)	
C3	0.6897 (4)	0.6859 (4)	1.0445 (3)	0.0249 (9)	
C4	0.7307 (4)	0.5650 (4)	0.9970 (3)	0.0269 (9)	
H4	0.6920	0.4807	1.0191	0.032*	
C5	0.8334 (4)	0.5746 (4)	0.9136 (3)	0.0261 (9)	
C6	0.8901 (5)	0.7016 (4)	0.8817 (3)	0.0325 (10)	
H6	0.9578	0.7048	0.8259	0.039*	
C7	0.8494 (5)	0.8233 (4)	0.9299 (3)	0.0348 (10)	
H7	0.8900	0.9075	0.9089	0.042*	
C8	0.8752 (5)	0.4488 (4)	0.8531 (3)	0.0280 (9)	
O1W	0.7018 (4)	1.1927 (3)	1.0612 (2)	0.0472 (8)	
H1A	0.7766	1.2210	1.0243	0.071*	
H1B	0.6896	1.2530	1.1073	0.071*	
O2W	1.2500	0.6297 (5)	0.7500	0.0456 (11)	
H2A	1.1720	0.5777	0.7590	0.068*	

### Atomic displacement parameters (Å^2^)

**Table:**

	*U*^11^	*U*^22^	*U*^33^	*U*^12^	*U*^13^	*U*^23^
Zn1	0.0350 (4)	0.0257 (4)	0.0303 (4)	0.000	0.0144 (3)	0.000
S1	0.0409 (7)	0.0283 (7)	0.0448 (6)	−0.0041 (5)	0.0230 (5)	−0.0092 (5)
O1	0.0392 (16)	0.0356 (18)	0.0363 (16)	−0.0035 (14)	0.0184 (13)	−0.0098 (14)
O2	0.0401 (16)	0.0301 (18)	0.0289 (15)	−0.0035 (13)	0.0129 (12)	−0.0031 (12)
N1	0.0341 (19)	0.0180 (18)	0.0353 (18)	−0.0021 (15)	0.0148 (15)	−0.0016 (15)
N2	0.0358 (19)	0.025 (2)	0.0323 (18)	−0.0004 (16)	0.0181 (15)	0.0008 (16)
C1	0.026 (2)	0.026 (2)	0.029 (2)	0.0005 (18)	0.0077 (17)	−0.0018 (17)
C2	0.026 (2)	0.022 (2)	0.030 (2)	0.0017 (17)	0.0081 (16)	−0.0006 (17)
C3	0.026 (2)	0.030 (2)	0.0206 (18)	0.0023 (17)	0.0088 (16)	0.0013 (17)
C4	0.029 (2)	0.023 (2)	0.030 (2)	0.0004 (17)	0.0091 (17)	0.0042 (17)
C5	0.028 (2)	0.031 (2)	0.0209 (19)	0.0029 (18)	0.0067 (16)	−0.0017 (17)
C6	0.033 (2)	0.038 (3)	0.029 (2)	−0.003 (2)	0.0162 (17)	0.003 (2)
C7	0.042 (2)	0.028 (3)	0.039 (2)	−0.0067 (19)	0.021 (2)	0.0044 (19)
C8	0.025 (2)	0.035 (3)	0.024 (2)	0.0016 (19)	−0.0012 (17)	−0.0022 (18)
O1W	0.064 (2)	0.0356 (19)	0.0453 (18)	−0.0104 (16)	0.0178 (15)	−0.0048 (15)
O2W	0.046 (3)	0.037 (3)	0.058 (3)	0.000	0.023 (2)	0.000

### Geometric parameters (Å, °)

**Table:**

Zn1—O2	1.987 (3)	C2—C3	1.400 (5)
Zn1—S1^i^	2.3159 (12)	C3—C4	1.374 (5)
S1—C1	1.707 (4)	C4—C5	1.400 (5)
O1—C8	1.254 (4)	C4—H4	0.9300
O2—C8	1.284 (5)	C5—C6	1.391 (5)
N1—C1	1.342 (4)	C5—C8	1.494 (5)
N1—C2	1.373 (5)	C6—C7	1.384 (6)
N1—H1	0.8600	C6—H6	0.9300
N2—C1	1.338 (5)	C7—H7	0.9300
N2—C3	1.392 (4)	O1W—H1A	0.84
N2—H2	0.8600	O1W—H1B	0.83
C2—C7	1.388 (5)	O2W—H2A	0.82
			
O2—Zn1—O2^ii^	102.22 (17)	C4—C3—N2	132.5 (4)
O2—Zn1—S1^i^	111.74 (8)	C4—C3—C2	122.2 (3)
O2^ii^—Zn1—S1^i^	114.66 (8)	N2—C3—C2	105.3 (3)
O2—Zn1—S1^iii^	114.66 (8)	C3—C4—C5	116.9 (4)
O2^ii^—Zn1—S1^iii^	111.74 (8)	C3—C4—H4	121.5
S1^i^—Zn1—S1^iii^	102.30 (6)	C5—C4—H4	121.5
C1—S1—Zn1^i^	103.48 (14)	C6—C5—C4	120.7 (4)
C8—O2—Zn1	115.9 (2)	C6—C5—C8	119.0 (3)
C1—N1—C2	109.0 (3)	C4—C5—C8	120.2 (4)
C1—N1—H1	125.5	C7—C6—C5	122.4 (3)
C2—N1—H1	125.5	C7—C6—H6	118.8
C1—N2—C3	109.5 (3)	C5—C6—H6	118.8
C1—N2—H2	125.3	C6—C7—C2	116.7 (4)
C3—N2—H2	125.3	C6—C7—H7	121.6
N2—C1—N1	108.8 (3)	C2—C7—H7	121.6
N2—C1—S1	128.2 (3)	O1—C8—O2	123.3 (4)
N1—C1—S1	123.1 (3)	O1—C8—C5	119.4 (4)
N1—C2—C7	131.5 (4)	O2—C8—C5	117.2 (3)
N1—C2—C3	107.4 (3)	H1A—O1W—H1B	107.0
C7—C2—C3	121.0 (4)		

Symmetry codes: (i) −*x*+1, −*y*+1, −*z*+2; (ii) −*x*+3/2, *y*, −*z*+3/2; (iii) *x*+1/2, −*y*+1, *z*−1/2.

### Hydrogen-bond geometry (Å, °)

**Table:**

*D*—H···*A*	*D*—H	H···*A*	*D*···*A*	*D*—H···*A*
N1—H1···O1W	0.86	1.88	2.738 (5)	174
N2—H2···O1^iv^	0.86	1.98	2.812 (4)	163
O1W—H1A···O2^v^	0.84	2.21	2.907 (4)	140
O2W—H2A···O1	0.82	2.02	2.837 (4)	177

Symmetry codes: (iv) *x*−1/2, −*y*+1, *z*+1/2; (v) *x*, *y*+1, *z*.
